# SARS-CoV-2 viral shedding characteristics and potential evidence for the priority for faecal specimen testing in diagnosis

**DOI:** 10.1371/journal.pone.0247367

**Published:** 2021-02-22

**Authors:** Chen Yuan, Hongling Wang, Kefeng Li, An Tang, Yaxin Dai, Bing Wu, Hui Zhang, Jiabei Chen, Jienan Liu, Wenjie Wu, Songye Gu, Hai Wang, Haodi Xu, Mingyu Wu, Menglu Yu, Yuchao Wang, Xinwei Yu, Jialu He, Shelan Liu, Yongli Zhang, Zhendong Tong, Jianbo Yan

**Affiliations:** 1 Department of Infectious Disease, Zhoushan Municipal Centre for Disease Control and Prevention, Zhoushan City, Zhejiang Province, People’s Republic of China; 2 Laboratory of Microbiology, Zhoushan Municipal Centre for Disease Control and Prevention, Zhoushan City, Zhejiang Province, People’s Republic of China; 3 Department of HIV/AIDS & Tuberculosis, Zhoushan Municipal Centre for Disease Control and Prevention, Zhoushan City, Zhejiang Province, People’s Republic of China; 4 HIV/AIDS Confirmation Laboratory, Zhoushan Municipal Centre for Disease Control and Prevention, Zhoushan City, Zhejiang Province, People’s Republic of China; 5 Department of Immunisation, Zhoushan Municipal Centre for Disease Control and Prevention, Zhoushan City, Zhejiang Province, People’s Republic of China; 6 Key Laboratory of Health Risk Factors for Seafood of Zhejiang Province, Zhejiang Province, People’s Republic of China; 7 Zhejiang Provincial Centre for Disease Control and Prevention, Hangzhou City, Zhejiang Province, People’s Republic of China; National Institute for Infectious Diseases Lazzaro Spallanzani-IRCCS, ITALY

## Abstract

This study aimed to identify the specimen type that has high positivity and its proper sampling time for severe acute respiratory syndrome coronavirus 2 (SARS-CoV-2) testing to promote diagnostic efficiency. All SARS-CoV-2-infected patients with a laboratory-confirmed diagnosis in Zhoushan City were followed up for viral shedding in respiratory tract specimens and faecal samples. Positivity was analysed both qualitatively and quantitatively by proper statistical approaches with strong testing power. Viral shedding in respiratory tract and faecal specimens was prolonged to 45 and 40 days after the last exposure, respectively. The overall positive rate in respiratory tract specimens was low and relatively unstable, being higher in the early-to-mid stage than in the mid-to-late stage of the disease course. Compared with respiratory tract specimens, faecal samples had a higher viral load, higher overall positive rate, and more stable positivity in different disease courses and varied symptomatic status. Faecal specimens have the potential ability to surpass respiratory tract specimens in virus detection. Testing of faecal specimens in diagnosis, especially for identifying asymptomatic carriers, is recommended. Simultaneously, testing respiratory tract specimens at the early-to-mid stage is better than testing at the mid-to-late stage of the disease course. A relatively small sample size was noted, and statistical approaches were used to address it. Information was missing for both specimen types at different stages of the disease course due to censored data. Our research extends the observed viral shedding in both specimen types and highlights the importance of faecal specimen testing in SARS-CoV-2 diagnosis. Healthcare workers, patients, and the general public may all benefit from our study findings. Disposal of sewage from hospitals and residential areas should be performed cautiously because the virus sheds in faeces and can last for a long time.

## Introduction

Coronavirus disease (COVID-19), caused by severe acute respiratory syndrome coronavirus 2 (SARS-CoV-2), has rapidly spread worldwide. The current epidemiological situation is not optimistic. As of January 9, 2021, more than 87.58 million cases and more than 1.90 million deaths have been reported globally [1], and several countries are facing new rounds of lockdown. By January 5, 2021, two different variants of SARS-CoV-2 have been reported in more than 40 countries/teritories/areas [2]. Generally, virus is not detectable until it is expelled from the host cell after successful replication, and the process is known as viral shedding. Fluorogenic real-time quantitative polymerase chain reaction (RT-qPCR) assay and genome sequencing are two techniques widely used in viral nucleic acid detection, and the former is usually preferred over the latter in SARS-CoV-2 testing and diagnosis. This is because the RT-qPCR assay overcomes the limitations of genome sequencing in aspects such as a high error rate, high technical requirements, insufficient reliability of antibody reagents, and low cost-effectiveness [3]. Lessons from other coronaviruses revealed that viral shedding duration and RT-qPCR assay testing positivity varied among different types of specimens [4–6]. However, limited information is known and no analysis has been conducted on SARS-CoV-2 [7–9]. Understanding the viral shedding pattern is helpful for timely and efficient diagnosis, which plays a decisive role in identifying the infection source, prompt isolation, conducting treatment, ending quarantine for the infected person, and disease prevention and control among the population.

We therefore aimed to identify the specimen type that has higher positivity and corresponding proper time of sampling, among commonly used respiratory tract and faecal specimens for SARS-CoV-2, to promote diagnosis efficiency. To achieve our goals, viral shedding patterns were observed, viral load was measured by Cycle threshold (Ct) values of RT-qPCR assay, and positive rates were analysed.

## Materials and methods

### Ethical approval

This study was approved by the internal ethics committee of Zhoushan CDC. By Law of the People’s Republic of China on Prevention and Treatment of Infectious Disease and relative regulations, all individuals within the territory of the People’s Republic of China must accept preventive and control measures of infectious diseases, such as investigation, inspection, sample collection, isolation and treatment [10]. Consent for sampling, testing and investigation from participants was exempted by law. No medical records were used for collecting information. All data had been fully anonymised before the transfer to the study group.

### Study design and case definition

A cross-sectional survey of baseline SARS-CoV-2 infection status was first conducted on all suspected person regardless of ethnicity and nationality from January 19 to March 2, 2020. To be more specific, our study included patients from the fever clinic, COVID-19 self-reported person, close contact of confirmed COVID-19 patients, and participants from surveillance programmes such as Influenza-Like Illness (ILI) and severe acute respiratory infection (SARI) in Zhoushan City. In order to enrol as many SARS-CoV-2 infected person as possible, no exclusion criteria was applied to the survey. Participants with equivocal test results and close contacts of known patients were kept in quarantine at the hospital and tested repeatedly. Then patients were followed up for observation of viral shedding in two types of biospecimens. Follow-up was initiated on the day of enrollment and terminated on March 2, 2020, or the last testing day with a negative result if the patient was tested negative on both 14 days and 28 days after discharge, whichever came first. According to the definition of China Centre for Disease Control and Prevention (China CDC), cases were defined as patients with confirmed diagnosis of SARS-CoV-2 infection by RT-qPCR assay [11]. Patients with samples of only one specimen type were excluded in further analyses because we focused on the comparison of the two specimen types.

### Sample collection

Respiratory tract specimens (i.e., nasopharyngeal swab and deep cough sputum), faeces, urine and serum specimens were collected. Inpatient specimens were collected by ward nurses, and specimens of other participants were collected by qualified CDC staff. All specimens were stored at 4°C for transportation and delivered to the laboratory in Zhoushan Municipal CDC within 2 hours. Multigelation (i.e., repeated freezing and thawing) of specimens was strictly prohibited.

### Nucleic acid extraction and PCR amplification

The device model ABI ViiA 7 Real Time PCR System and corresponding software were used. Molecular testing kits, article number SJ-HX-226-1,2, manufactured by Shanghai BioGerm Medical Biotechnology Co., Ltd, were used for all tests. Viral RNA was extracted from the collected specimens (150 μl) using a Viral RNA Mini Kit (Qiagen, article number 74104, Germany) according to the manufacturer’s protocols. The nucleic acid was manually added into the reaction system (RT-qPCR reaction fluid, 12 μl; RT-qPCR enzyme mixture, 4 μl; and SARS-CoV-2 primer probe, 4 μl). After instantaneous low-speed centrifugation, the mixture was added to the RT-qPCR device for amplification. The mixture was first reverse transcribed through the following steps: 50°C for 10 min for 1 cycle; pre-denaturation at 95°C for 5 min for 1 cycle; then denaturation at 95°C for 10 s and annealing/extension at 55°C for 40 s repeated for 40 cycles. Pre-treatment, RNA extraction, and PCR amplification were completed within 4 hours after sampling to minimise molecular decomposition. All specimens were processed in a biosafety level-2 laboratory, and all procedures except for PCR amplification were carried out in biological safety cabinets. Personnel used protective measures such as an N95 mask (3M, type number 1860), a surgical cap, goggles, medical protective clothing, shoe cover, and latex gloves. All laboratory wastes were treated as infectious medical wastes, disposed into specific medical garbage bags, and then sterilised in autoclaves.

### Data analyses

For different specimen types, positive rates were calculated as the number of positive samples divided by the number of all tested samples among the whole tested population and cases. The suspected exposure date was investigated retrospectively for cases. The following age groups were considered in this study using the criterion of China Information System for Disease Control and Prevention: 0–15, 16–25, 26–45, 46–65, and 66-maximum. Participants with fever, running nose, nasal congestion, cough, and sneeze were categorised as being symptomatic. Pearson’s chi-square tests with/without Yate’s continuity correction were applied based on the eligibility to explore the association between positivity and covariates, such as age, sex and symptomatic status, among the study population for both specimen types. Viral shedding duration was defined as the timespan of viral shedding in units of days and was calculated as the last date with a positive RT-qPCR result minus the first date with a positive RT-qPCR result plus 1. A positive RT-qPCR result meant either a Ct value of the ORF1ab region <40 or a Ct value of the N region <40 in the RT-qPCR assay. Furthermore, the difference in viral shedding duration between respiratory tract and faecal specimens was analysed using Wilcoxon signed-rank test. The Ct values of RT-qPCR assay were analysed using Wilcoxon rank-sum test. Moreover, test results of varied specimens collected on the same date of the same case were recorded, and their consistency was analysed using McNemar’s chi-square test with continuity correction.

Finally, time value was added to illustrate positivity among varied specimen types in different disease courses. Cumulative positive rates were measured to diminish the unsteadiness of representativeness caused by a small test frequency of the RT-qPCR assay. To be more specific, the cumulative positive rate of day X (as the day after the last exposure) backward was calculated as cumulative positive test frequency from day 1 to day X divided by cumulative total test frequency from day 1 to day X. Moreover, cumulative positive rate of day X forward was calculated as cumulative positive test frequency from day (X+1) to the last testing day divided by cumulative total test frequency from day (X+1) to the last testing day. For every single day, cumulative positive rate of day backward and that of day forward were calculated and further compared as paired data using Wilcoxon signed-rank test. The comparisons were repeated for both respiratory tract specimens and faecal samples. R (The R Foundation, version 3.6.3) and its package {ggplot2} (version 3.2.1) were used for data analyses and visualisation. Two-sided testing was applied to all statistical analyses, and P<0.05 was considered statistically significant.

## Results

### Analyses of the whole study population

From January 19 to March 2, 2020, a total of 1,384 participants were enrolled and 2,148 specimens were tested. Positive signals were detected in 29 of 1,977 respiratory tract specimens (1.47%) and 51 of 171 faecal specimens (29.82%). No urine or serum specimen was positive (n = 8 and n = 12, respectively). Equivocal results of 9 respiratory tract specimens and 1 faecal specimen (of 6 different individuals) were reported, and in fact, the corresponding participants all had a confirmed diagnosis based on tests conducted on other dates. Notably, specimens collected from 117 participants from the ILI surveillance programme and 18 from the SARI surveillance programme testing for both SARS-CoV-2 and influenza (A/H3N2, A/H1N1, B) reported no co-infection of SARS-CoV-2 and influenza. Each of the 1,384 participants had at least one respiratory tract specimen and one faecal sample tested. As shown in Table 1, the age range of our participants was 0–103 years, with a median age of 36 years. The male-to-female ratio was 1.02, and 64.38% of them had signs such as fever, running nose, and cough. Chi-square tests revealed that age group was significantly associated with positive rates of SARS-CoV-2 infection, for both specimen types (P = 0.002 and <0.001 for respiratory tract and faecal specimens, respectively). Meanwhile, asymptomatic participants had a lower positive rate for respiratory tract specimen than symptomatic participants (P = 0.005), whereas the positive rate of faecal samples did not show any significant difference by symptomatic status (P = 0.295). Finally, sex was not statistically significantly associated with SARS-CoV-2 positivity in either specimen type (P = 0.389).

**Table 1:** Distribution of positivity for the 1,384 participants.

	Total (N = 1,384)	Negative (n = 1,374)	Positive (n = 10)
**Age, years**			
**0–15**	132	130	2
**16–25**	138	138	0
**26–45**	639	634	5
**46–65**	348	346	2
**66–103**	127	126	1
**Sex**			
**Male**	699	694	5
**Female**	685	680	5
**Symptomatic status**			
**Symptomatic**	891	885	6
**Asymptomatic**	493	489	4

Participants with fever, running nose, nasal congestion, cough, and sneeze are categorised as being symptomatic.

### Description of cases

**Fig 1:**
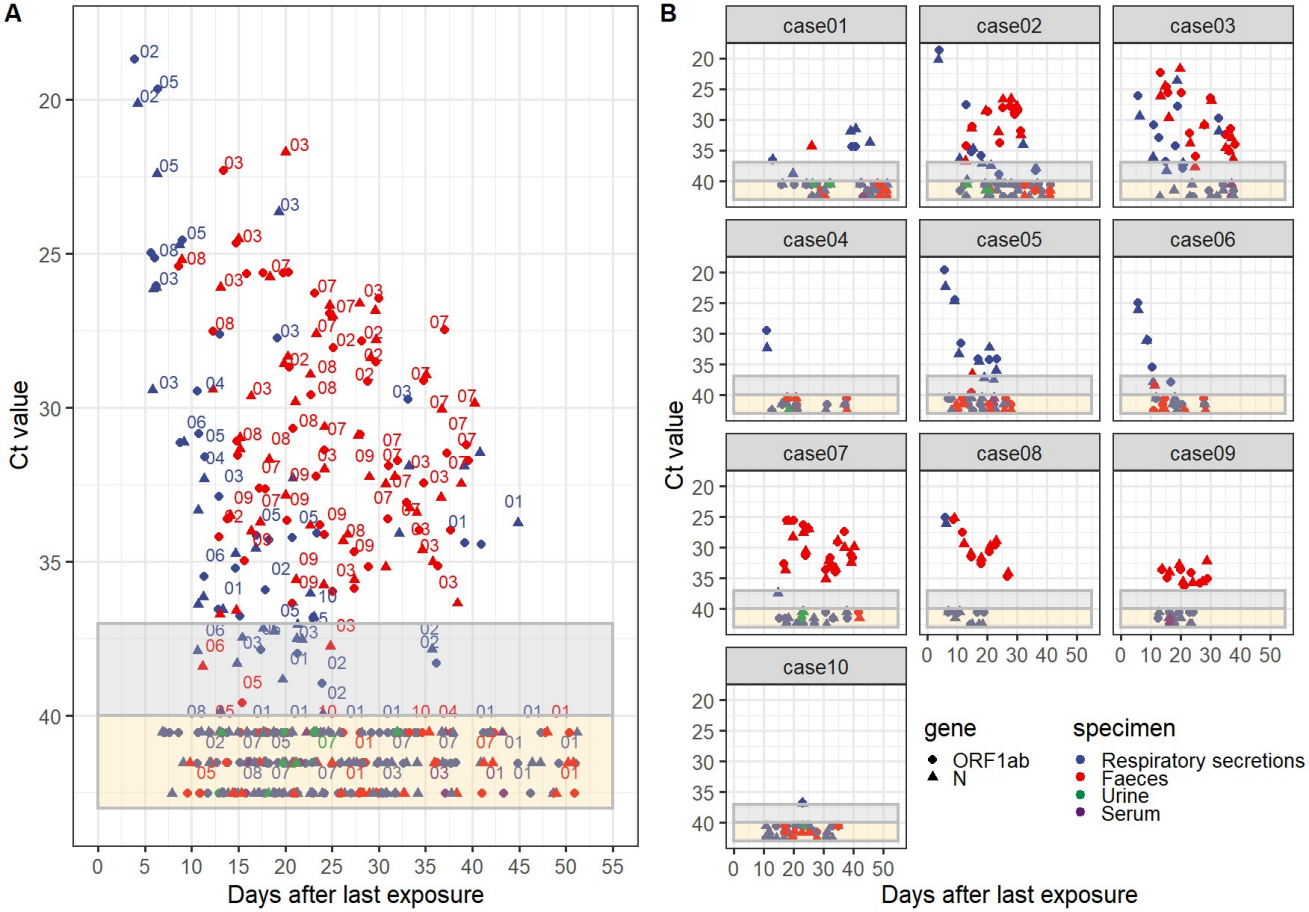
Cycle threshold (Ct) values of fluorogenic real-time quantitative polymerase chain reaction (RT-qPCR) assay targeting the ORF1ab and N regions of SARS-CoV-2 on days after the last exposure for 10 independent cases. Marks in the grey area indicate specimens with equivocal results, and those in the yellow area indicate specimens with negative results. Negative specimens with no accurate Ct values were assigned to random numbers from 40 to 43 only for better visualisation. **A**, Gathered scatter plot. Numbers on the upper right or top of the marks represent individual case number. Different shapes represent the ORF1ab and N genes respectively and distinct colours represent specimen types, namely, respiratory secretions, faeces, urine and serum. B, Individual scatter plot.

By the time our study initiated, no known case had been reported previously. During the whole study period, only 10 participants met our criteria as cases; their viral shedding characteristics in different specimen types are illustrated in Fig 1 and Table 2. These 10 cases were followed up for 12–39 (median 23.5) days for viral shedding in respiratory tract secretion and 16–29 (median 20) days for viral shedding in faecal samples. No one dropped out of the follow-up. Of these 10 cases, all were Chinese, 5 were male, 4 were asymptomatic, and their age ranged from 7 to 68 (median 41) years. The frequency of repeated specimens from each case varied from 6 to 21 (median 10.5) and 3 to 15 (median 7), for respiratory tract and faecal specimens, respectively. Viral shedding in respiratory tract specimens lasted for 1–33 (median 12) days and was still detectable on days 6 to 45 (median day 23) after the last exposure. Viral shedding in faeces lasted for 1–26 (median 17.5) days, and it was still detectable on days 11 to 40 (median day 28) after the last exposure. Moreover, the difference in the observed viral shedding duration between two specimen types was not statistically significant (P = 0.688). Of note, we discovered 1 case with recurrent positive results in respiratory tract specimen after discharge, and 3 cases with positive results in faecal samples in their final tests before discharge on the last observation day (Table 2).

**Table 2:** Viral shedding characteristics in different specimen types for 10 cases of SARS-CoV-2 infection.

Case	Sex	Age, years	Symptomatic status	Day of follow-up (n)		Frequency of specimen (n)			Detectable duration of viral shedding (days)		Observed on the last day (after the last exposure) of viral shedding	
				Respiratory tract specimen	Faecal specimen	Subtotal	Respiratory tract specimen	Faecal specimen	Respiratory tract specimen	Faecal specimen	Respiratory tract specimen	Faecal specimen
**Case 01**^	Male	42	Symptomatic	39	26	23	16	7	33	1	45	26
**Case 02**	Male	65	Symptomatic	38	29	34	21	13	33	19	36	31
Case 03*	Female	68	Symptomatic	33	26	26	14	12	28	26	33	38
**Case 04**	Male	34	Symptomatic	27	21	11	8	3	1	NE	11	NE
**Case 05**	Female	65	Symptomatic	21	19	21	15	6	18	1	23	15
**Case 06**	Female	43	Symptomatic	23	18	15	10	5	12	1	17	11
**Case 07**	Male	10	Asymptomatic	24	26	24	9	15	1	24	15	40
Case 08*	Female	7	Asymptomatic	14	19	14	7	7	1	19	6	27
Case 09*	Male	36	Asymptomatic	12	16	13	6	7	NE	16	NE	29
**Case 10**	Female	40	Asymptomatic	23	19	18	11	7	1	NE	23	NE

Duration of viral shedding is calculated as the time lag from the first to the last day when the cycle threshold (Ct) value of the ORF1ab or N region was <40.

Note: NE, molecular diagnosis result is negative.

*, patient was discharged from the hospital with a positive faecal specimen and isolated in a designated setting.

^^^, patient with recurrent positive results in respiratory tract specimens after discharge.

### Analyses of positivity in two specimen types among 10 cases

**Fig 2:**
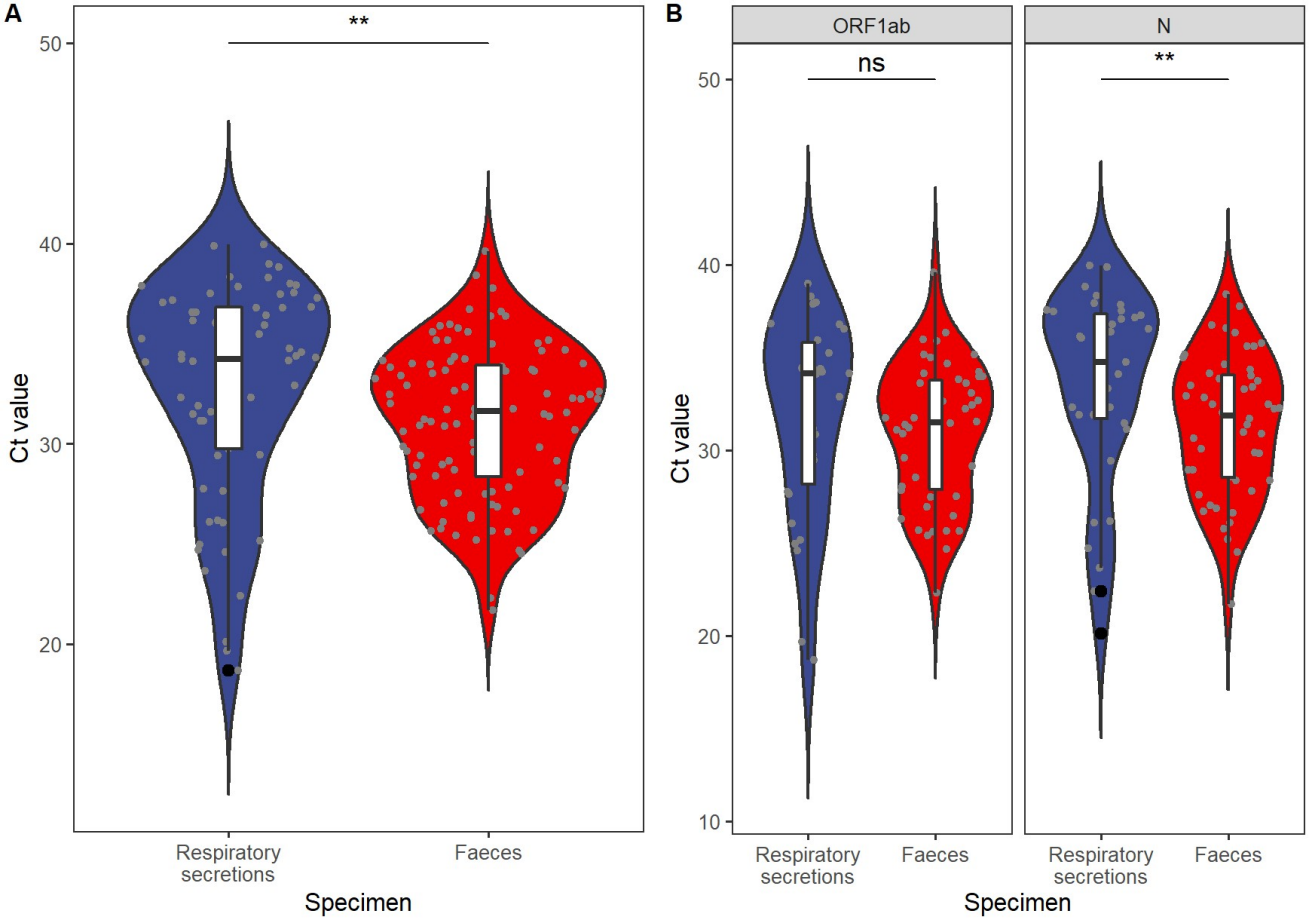
Differences in Cycle threshold (Ct) values between specimens of respiratory secretions and faeces in 10 independent cases. The boxes and whiskers show the median, interquartile, and full range of Ct values. Boxes in blue represent respiratory secretions, and boxes in red represent faecal specimen. Jittered grey dots represent specimens with corresponding Ct values, and signs on the top illustrate statistical significance. **, statistically significant on level of 0.001<P<0.01; ns, P>0.05. **A**, Overall comparison with Ct values in both the ORF1ab and N regions. With P = 0.002 analysed by Wilcoxon rank-sum test, the Ct value of faecal samples is lower than that of respiratory secretions; hence, the viral load is higher in faecal specimens than in respiratory secretions. **B**, Comparison for each individual targeted region of SARS-CoV-2. With P = 0.006 analysed using Wilcoxon rank sum test, the Ct value of faecal specimens is lower than that of respiratory secretions for the N region.

**Fig 3:**
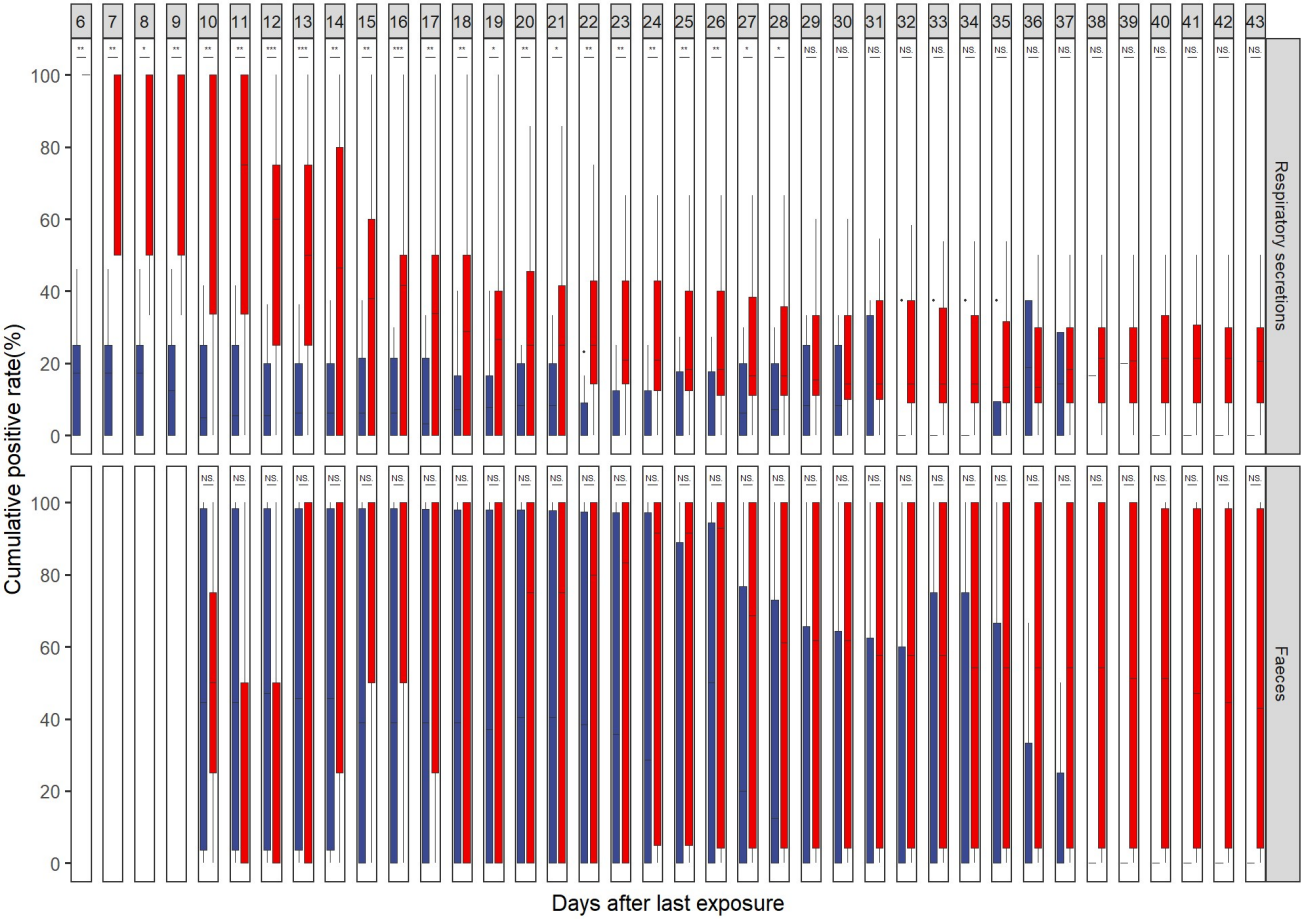
Cumulative positive rates of specimen collected on days backward/forward after the last exposure from SARS-CoV-2 infections for 10 independent cases. The boxes and whiskers show the median, interquartile and full range of cumulative positive rates. Boxes in red represent cumulative positive rates of days backward (cut-off value included), and boxes in blue represent rates of days forward (cut-off value excluded). ***, statistically significant on a level of 0.0001<P<0.001; **, statistically significant on a level of 0.001<P<0.01; *, statistically significant on a level of 0.01<P<0.05; ns, P>0.05. For respiratory secretions, cumulative positive rates are continually higher in the first 6th to 28th days (as the day after the last exposure) backward compared with cumulative positive rates on the corresponding day forward, with P values of 0.006, 0.006, 0.014, 0.006, 0.002, 0.002, 0.0009, 0.0009, 0.002, 0.002, 0.0008, 0.002, 0.005, 0.013, 0.0099, 0.02, 0.001, 0.003, 0.003, 0.005, 0.005, 0.041, and 0.041 corresponding to days 6–28. No statistically significant difference is found in faeces. All analyses were based on the method of Wilcoxon signed-rank test.

Among these 10 cases, 29 of 117 respiratory tract specimens were reported positive, and the positive rate was 24.79%. Likewise, 51 of 82 faecal specimens were tested positive, making the positive rate as high as 62.20%. Ct values of the ORF1ab and N regions from respiratory tract specimens were higher than those from faecal specimens (P = 0.002, Fig 2). More specifically, it was the N region that Ct values differed by specimen types (P = 0.006). Additionally, with poor consistency among two specimen types, McNemar’s test with continuity correction suggested faecal specimens had a higher positive rate than respiratory tract specimens (McNemar’s χ^2^ = 12.19, P<0.001, Kappa = 0.034, Table 3). Finally, cumulative positive rates of day X backward were suggested to be higher than those of day X forward for respiratory tract specimens from day 6 to day 28 (i.e. 6≤X≤28), with P values ranging from 0.0008 to 0.041. Conversely, no statistically significant difference was identified in any day for cumulative positive rates of day backward/forward comparison for faecal specimens (Fig 3).

**Table 3:** Consistency of test results between different specimens of the same case collected on the same date.

		**Faeces**		**Total**
		+	-	
**Respiratory secretions**	+	4	2	6
	-	19	13	32
**Total**		23	15	38

With McNemar’s χ^2^ = 12.19 and P<0.001, the positive rate in faecal specimens is higher than that of respiratory tract specimens, and the difference is statistically significant. A Kappa value of 0.034 suggests poor consistency of results between two specimen types, which strengthens the superiority of faeces in diagnosis.

## Discussion

### Key results

In this study, we report several meaningful discoveries. To start with, the Ct values of respiratory tract specimens by RT-qPCR assay were higher than those of faecal specimens for the whole observed disease course. With the reverse relationship between Ct value and viral load, we reported a higher viral load for faecal specimens than that for respiratory tract specimens. Additionally, McNemar’s chi-square test with continuity correction suggested a higher positive rate in faecal specimens than in respiratory tract specimens for the whole observation period. These findings suggest that faecal specimens surpass respiratory tract specimens in virus detection in our study. Moreover, faecal positivity did not vary by symptomatic status or disease course. Together with the fact that the virus remained detectable in faeces on day 40 from the last exposure, we recommend testing of the faecal specimen during diagnosis, especially for identifying asymptomatic carriers in disease surveillance. As for respiratory tract specimens, viral shedding prolonged to day 45 after the last exposure. Higher cumulative positive rates of specimens collected on continuous days 6–28 (inclusive) backward than those collected on the corresponding day forward suggest that the best sampling time is during days 6–28 after the last exposure, which occurs during the early-to-mid stage of the disease course. Therefore, testing of respiratory tract specimens in the early-to-mid stage is better than testing at the mid-to-late stage of the disease course.

### Interpretations

To the best of our knowledge, studies of SARS-CoV-2 are limited, and the findings of the present study are novel. Published literature mainly focused on transmission dynamics, epidemiological features, clinical characters, genomics, psychology, and comments on COVID-19 [12], and a few reported positivity and viral shedding pattern [13]. Our finding is in agreement with a documented positive rate of 48.1%-66.67% in faecal samples [14–16] and is slightly lower than the positive rate of 29.6%-61.3% among throat swabs reported previously [17, 18]. With regard to the viral shedding pattern, despite an incubation period of 1–14 days [19], our observed longest viral shedding on day 45 after the last exposure in respiratory tract specimens and for a duration of 26 days in faecal samples are still much longer than the previously reported viral shedding of day 24 after disease onset in nasopharyngeal aspirates and for a duration of 1–7 days in faecal samples [20]. Simultaneously, lately published data suggest a lower Ct value in rectal swabs than in nasopharyngeal swabs among children under 15 years old [21]. Our finding is in line with their opinion but in a different age group, and we strengthen our opinion by scientific strategies. All these progresses we made led to a step towards better understanding of the novel virus and are of practical meaning in clinical diagnosis and public health perspectives. Whether the SARS-CoV-2 viral shedding pattern resembles that of SARS-CoV, declining rapidly in the respiratory tract after a peak and can be prolonged to over two months in the gastrointestinal tract [4, 22], further studies with a larger sample size and a longer observation period are needed.

Interestingly, we noticed that a lower viral load in faecal specimens (550–1.21 x 10^5^ copies per mL) was reported than that in respiratory tract specimens (641–1.34 x 10^11^ copies per mL) [23]. As no statistical analysis was performed, this difference might be explained by sampling error. In fact, positivity of virus detection can be interfered by many factors. First, location and operation of sampling may affect virus detection. As mentioned previously, the viral load differs by location, and inappropriate procedure may lead to missing of viral clusters with high concentration. Our specimens were collected by trained nurses and qualified CDC staff under a standard protocol; therefore, the sampling error in biosamples collected from each individual was reduced. Next, storage condition after sampling is also concerning. If placed at an inappropriate temperature or for a long time, then the naturally unstable RNA will be degraded. In our study, all specimens were stored at 4°C, pre-treated and tested within 4 hours upon sampling; thus, the false-negative result caused by decomposition of the SARS-CoV-2 molecular structure was minimised. Finally, laboratory procedures and materials also play important roles, and the possibility of cross-reaction between SARS-CoV-2 and other viruses with similar genome sequences should be alerted. In our study, all tests were conducted in the same laboratory under the same condition by the same qualified personnel; therefore, measurement bias was low. Furthermore, all specimens were tested using commercial kits from the same company based on China CDC’s primer-probe set and of the same batch number; then consistency was assured. However, due to the sudden outbreak, the research and development phase of this testing kit was relatively short and the kit was presumably verified in limited sample size in the clinical trial; then there is a possibility that product quality might have been compromised. For records, China CDC’s primer-probe set targeting the ORF1ab gene was verified as the most sensitive set compared with sets developed by institutions from Germany and Hong Kong, but its performance targeting the N region was inferior to that of Japan and the USA [24]. For all these reasons, RT-qPCR results should be interpreted with caution.

### Implications

Together with the evidence that sometimes SARS-CoV-2 RNAs were not detectable among any type of upper respiratory tract specimens even in severe cases [18], we highlight the importance of faecal specimen testing in SARS-CoV-2 diagnosis. From the perspective of clinical practice, our findings help to facilitate efficient diagnosis and reduce the risk for healthcare workers at sampling by minimising the possibility of respiratory droplets formation, which is believed to be the main transmission route by the World Health Organization [19]. As there is shortage of the personal protection equipment, our findings provide a simple and efficient way to protect understaffed healthcare workers. In the interest of the object being sampled, collection of the excreted faeces produces no discomfort, whereas being inserted a swab into the nasopharynx is not pleasant; therefore, choosing to collect faecal samples may enhance the compliance of patients. From the perspective of public health, given that faecal samples surpass respiratory tract specimens in identifying asymptomatic carriers who are infectious and may be a source of transmission [25], testing of faecal specimens in diagnosis is of significance in disease control and prevention for the population. Finally, our finding of prolonged viral shedding in faeces raise concerns on disposal of sewage, especially for relevant municipal stakeholders who are responsible for dealing with household waste from self-quarantined individuals and anonymous asymptomatic carriers. Sewage shall be disinfected appropriately before discharge into the environment.

### External validity

In this study, we have analysed all SARS-CoV-2-infected patients from January 19 to March 2, 2020 in Zhoushan City. We have also presented viral shedding characteristics and potential evidence revealing the importance of faecal specimen testing regardless of symptomatic status and disease course. Due to the government’s quick and effective response to the disease, we have a limited number of infected patients. This weakens external validity, and our findings may better apply to cities with similar acreage and population. To be more specific, Zhoushan city has an acreage of 1,459 square kilometres. The population was 1.173 million and its density was 804 person/square kilometres at the end of 2019 [26]. With regard to overcrowded international metropolises, our findings might serve as an instructive hypothesis and we hope further studies could be conducted to support or oppose our findings.

### Limitations

Like all studies, our study has limitations. First, the total number of cases was relatively small. To overcome this difficulty, we used rates of specimen instead of rates of person in description. Moreover, cumulative positive rates were adopted to address the unsteadiness of representativeness caused by a small RT-qPCR assay test frequency. In addition to measurements, most of our comparisons were designed as paired data, and proper statistical strategies with strong testing power were chosen. Second, we missed some information on viral shedding in both specimen types at different stages of the disease course. Because the novel SARS-CoV-2 was newly discovered, very limited information was known about it at the time we started our research. Initially, we did not know that the virus may shed through faeces, and consequently, no faecal specimen was collected in the first few days. At the same time, we reported 1 case with recurrent positive results in respiratory tract specimens after discharge and 3 cases with positive results in the faeces on the day the observation terminated as censored data. These led to the underestimation of viral shedding duration in both specimen types. Third, no lower respiratory tract specimen was collected because no invasive ventilation was applied to any participant. As major damage occurs in the lung and lower respiratory tract [27–29], specimens from the upper respiratory tract, which comprises nose, nasal cavity, mouse, pharynx, and larynex [30], presumably have a low viral load. Hence, the possibility of underreporting on positivity in respiratory tract specimens exists. Fourth, information of baseline health condition and antiretroviral treatment was not obtained, then their impact on viral shedding was not known. Fifth, as four patients were asymptomatic, we chose the day after the last exposure for measurement instead of the day after disease onset. This measurement is of public health importance but affects comparison with the data of other clinical studies. Lastly, the Ct value used to express the viral load is inferior to virus copy number in accuracy due to algebraic manipulation and is subject to threshold and baseline selection in device operation.

## Conclusions

With limited sample size and some censored data, we present potential evidence of the importance of faecal specimen detection regardless of symptomatic status and disease course in our study. Through preliminary description and comparison, viral shedding patterns in respiratory tract and faecal specimens are partially revealed, and the proper sampling time for each specimen type is provided. In view of the fact that the COVID-19 pandemic has not been eased, our findings can help to improve the efficacy of diagnosis. Overall, accurate and sensitive diagnosis in the early stage is of great significance in treatment for individuals and is crucial in disease surveillance, control, and prevention for the population. Future studies may work on larger population and observe viral shedding in faeces in different races and ethnicities.

## Supporting information

S1 Data (CSV)
